# Antidiabetic Effects of the Ethanolic Root Extract of *Uvaria chamae* P. Beauv (Annonaceae) in Alloxan-Induced Diabetic Rats: A Potential Alternative Treatment for Diabetes Mellitus

**DOI:** 10.1155/2018/1314941

**Published:** 2018-11-08

**Authors:** Jonathan Emeka Emordi, Esther Oluwatoyin Agbaje, Ibrahim Adekunle Oreagba, Osede Ignis Iribhogbe

**Affiliations:** ^1^Department of Pharmacology and Therapeutics, College of Medicine, Ambrose Alli University, Ekpoma, Nigeria; ^2^Department of Pharmacology, Therapeutics and Toxicology, College of Medicine, University of Lagos, Lagos, Nigeria

## Abstract

Diabetes mellitus has been a menace to mankind from time immemorial. However, a natural product such as *U. chamae* P. Beauv (Annonaceae) offers alternative treatment for diabetes mellitus. The study aimed at evaluating antidiabetic activity of the ethanolic root extract of *U. chamae* in alloxan-induced diabetic rats. Diabetes was induced in Sprague Dawley rats after overnight fast with 150 mg/kg alloxan intraperitoneally. After 72 h, those with plasma glucose levels >200 mg/dl were classified as diabetic. Five diabetic rats in each group were treated daily for 14 days orally with 100, 250, and 400 mg/kg of the extract, glibenclamide (71 *µ*g/kg) and pioglitazone (429 *µ*g/kg), respectively, while another group was untreated. Control received 0.5 ml of *Acacia senegal*. Effects of extract on glucose, other biochemical, and hematological parameters were evaluated. *α*-amylase and *α*-glucosidase inhibitory activities of extract and its fractions were also evaluated. Percentage inhibition and IC_50_ values were determined. Diabetic control was achieved on the 7th day of the study with 100, 250, and 400 mg/kg of the extract showing glucose reduction of 72.14%, 78.75%, and 87.71%, respectively. The HDL-cholesterol levels of diabetic rats treated with extracts were significantly increased. Extract and its fractions caused *α*-amylase and *α*-glucosidase inhibition. Histologically, pancreas of diabetic rats treated with extract showed regenerated islet cells which were not seen in rats treated with glibenclamide and pioglitazone. This study showed that *U. chamae* has antidiabetic activity which may be through *α*-amylase and *α*-glucosidase inhibition and regeneration of pancreatic beta cells. Also, it may reduce the risk of cardiovascular disease by increasing HDL-cholesterol levels.

## 1. Introduction

Diabetes mellitus (DM) has been a threat to mankind from time immemorial, and it is now wreaking havoc disproportionately worldwide [1]. It is a public health problem acknowledged as one of the most important killer diseases and a prominent cause of death in low- and middle-income countries [2]. The life expectancy of diabetic patients is usually low compared to normal people [3]. DM is a noncommunicable disease in which there is a metabolic disorder of various etiologies described by sustained hyperglycemia with disorders of carbohydrate, fat, and protein metabolism following defects in insulin secretion, insulin action, or both [4]. It is caused by the destruction of pancreatic *β*-cells or dysfunctional *β*-cell and insulin resistance which results in hyperglycemia [5, 6]. Over time, diabetic patients with poor glycemic control undergo micro- and macrovascular complications including nephropathy, retinopathy, neuropathy, and cardiovascular diseases [7]. These complications increase their suffering and are the major sources of expenses for patients with diabetes as well as increasing the financial burden of nations [8, 9]. Above and beyond insulin are other therapeutic options for the treatment of type 1 diabetes which include transplantation of whole organ pancreas and isolated islets, marred by both lack and quality of the donor's pancreas [10, 11]. However, numerous agents that are currently used for the treatment of type 2 diabetes are facing limited efficacy and tolerability [12]. For instance, sulfonylureas induce *β*-cell death in isolated rodent and human islets while glucagon-like peptide-1 receptor agonists and dipeptidyl peptidase-4 inhibitors have potential risks for pancreatitis, pancreatic, and thyroid cancers [13–15]. Therefore, logical long-term solution to diabetic therapy is restoration of *β*-cells since *β*-cell deficiency underlies both type 1 and type 2 diabetes [16]. The restoration of deficient *β*-cell mass by transplantation from exogenous sources or by endogenous regeneration of insulin-producing cells would undoubtedly be a worthwhile therapeutic goal that will significantly ameliorate diabetes and its complications [17, 18]. Another approach to the treatment of diabetes is the application of medicinal plants with phytochemicals that cause beta-cell regeneration leading to normal blood glucose in animals and humans [19]. Many medicinal plants of African origin, such as *Momordica charantia* (bitter melon), *Cyclopia genistoides* (honeybush), and *Catharanthus roseus* (Madagascan periwinkle), are effective against various diseases including diabetes mellitus [20]. *Uvaria chamae* is one of such plants used traditionally to treat diabetes mellitus and other conditions such as bronchitis, gastroenteritis, amenorrhea, menorrhagia, abdominal pain, and wound healing [21–23]. It is a climbing medicinal plant that belongs to the family Annonaceae and is commonly found in West Africa, where it is known with different names by the Igbo, Hausa, Yoruba, Esan, and Igala natives of Nigeria as *Mmimi ohia*, *Kaskaifi*, *Oko oja*, *Ogholo*, and *Ayiloko*, respectively [24]. Several studies have confirmed that the bioactive compounds of *U. chamae* such as alkaloids, flavonoids, phenols, tannins, and terpenoids produce hypoglycemic, anti-inflammatory, antifungal, and antimalarial effects [24–27]. However, there is limited documentation on the potential use of *U. Chamae* in the treatment of diabetes mellitus. Therefore, this study aimed to evaluate antidiabetic effects of ethanolic root extract of *U. chamae* in alloxan-induced diabetic rats and its potential use in the treatment of diabetes mellitus.

## 2. Materials and Methods

### 2.1. Collection and Extraction of *Uvaria chamae*

The roots of the plants were collected in the Esan Central region of Edo state, Nigeria. They were identified and authenticated by Mr. T. K. Odewo, a taxonomist in the Department of Botany and Microbiology, Faculty of Science, University of Lagos, Nigeria. The voucher specimen numbered LUH 3572 was deposited in the institutional herbarium. The plant extraction was done using the methods described by Emordi et al. [23]. The ethanolic root extract of *U. chamae* (crude) was separated into chloroform, ethyl acetate, and ethanolic fractions via column chromatography.

### 2.2. Animals for the Experiment

The thirty-five animals used in this study were 6–8-week-old Sprague Dawley rats of either sex weighing 160 ± 20 g acquired from the Animal Center, College of Medicine, University of Lagos, Idi-Araba, Lagos State, Nigeria. They were placed into 7 groups of 5 rats and maintained under standard environmental condition (12/12 hr light/dark cycle) with free access to water and standard rodent diet (Pfizer Feeds Plc., Nigeria). The cage beddings and water bottles were cleaned daily, and the animals were allowed to adapt for two weeks to the laboratory conditions before the beginning of the experiment.

### 2.3. Ethical Considerations

The experimental protocol was approved by the Research grants and Experimentation Ethics Committee on animal use of the College of Medicine, University of Lagos, Lagos, Nigeria (with a protocol ID:RGEEC/21/2015). This was carried out in strict compliance with the National Research council guidelines on the care and use of laboratory animals [28].

### 2.4. Experimental Procedures

#### 2.4.1. Induction of Diabetes

Except for the rats in group 1 (control), DM was experimentally induced in the animals of groups 2–7, after fasting them overnight by intraperitoneal administration of alloxan monohydrate dissolved in normal saline (150 mg/kg) [29]. Three days later, the blood glucose measurements were monitored with a glucometer, and the rats with plasma glucose greater than 200 mg/dl were labeled diabetic [23].

#### 2.4.2. Animal Treatment

The treatment of the animals via oral route lasted for 14 days. Group 1 (normal control) received 0.5 ml (2% solution) of *Acacia senegal*. Groups 2 and 3 received 71 *µ*g/kg of glibenclamide and 429 *µ*g/kg of pioglitazone, respectively. Groups 4, 5, and 6, received 100, 250, and 400 mg/kg of the root extract of *U. chamae*, respectively, as determined by the outcome of the acute toxicity study by Emordi et al. [23]. Group 7 was not treated with the extract as it represented the diabetic control. During the treatment period, the weight of the animals and the fasting blood glucose (FBG) measurements were determined with a weighing scale and a glucometer (using the tail vein), respectively, every 2 days from the beginning of the treatment (day 1) to the last day of the experiment (15th day).

#### 2.4.3. Sample Analysis

The blood was collected on the 15th day through ocular puncture into heparinized bottles for biochemical assays, ethylenediaminetetraacetic acid (EDTA) bottles for hematological assays, and plain bottles for insulin assay and the rats sacrificed. The blood samples with anticoagulants were centrifuged within five minutes of collection for 10 min at 4,000 g. By precipitation and modified enzymatic procedures from Sigma Diagnostics, the total cholesterol (TChol), triglyceride [30], and high density lipoprotein- (HDL-) cholesterol measurements were determined from the obtained plasma while the Friedewald equation was used to calculate low-density lipoprotein- (LDL-) cholesterol [31]. Also, creatinine and the enzymes (aspartate aminotransferase [32], alanine aminotransferase (ALT), and alkaline phosphatase (ALP)). obtained from the plasma were evaluated using standard enzymatic assay methods [33]. Additionally, the plasma glucose, total protein, and albumin levels were determined using enzymatic spectroscopic methods [34].

#### 2.4.4. Histological Studies

At the end of the experiment, the animals were sacrificed, and vital organs including the pancreas were harvested and fixed in 10% buffered formalin. The pancreatic tissue was processed using standard procedures as described by Grizzle et al. [35]. The tissue section was observed with a light microscope at a high magnification for histological changes and photomicrographs taken.

### 2.5. Determination of *α*-Amylase Inhibition

The determination of *α*-amylase inhibition by *U. chamae* was carried out according to the modified method by Kazeem et al. [36]. The mixture containing 200 *μ*l of 0.02 M sodium phosphate buffer (pH 6.9), 20 *μ*l of alpha-amylase, and 200 *μ*l of the plant extract or its fractions in a concentration of 10–100 *μ*g/ml was incubated for 10 minutes at 37°C, followed by addition of 200 *μ*l of 1% starch solution in all the test tubes. The mixture was incubated for 15 min at 37°C. Addition of 400 *μ*l dinitrosalicylic acid (DNS) reagent was used to terminate the reaction. The mixture was placed in a boiling water bath for 5 minutes, cooled, and diluted with 5 ml of distilled water, and the absorbance measured at 540 nm. The control samples were prepared without any plant extracts. The % inhibition was calculated according to the following formula:

(1) $$\begin{array}{c} \text{inhibition}\;\;\left(\text{\%}\right)=\text{Abs}\;\;540\;\;\left(\text{control}\right)-\text{Abs}\;\;540\;\;\left(\text{extract}\right)\times \frac{100}{\text{Abs}\;\;540\;\;\left(\text{control}\right)}. \end{array}$$

The IC_50_ values were calculated by nonlinear regression analysis from the mean inhibitory values. Acarbose (STD = standard) was used as the reference *α*-amylase inhibitor. All tests were performed in triplicate.

### 2.6. Determination of the Type of *α*-Amylase Inhibition

The determination of the type of *α*-amylase inhibition by *U. chamae* and its fraction was done using the crude extract of *U. chamae* and its chloroform fraction that had the lowest IC_50_. The experiment was carried out according to the modified method described by Kazeem et al. [36]. The extract and its chloroform fraction (250 *μ*l of 5 mg/ml) were placed in two sets of test tubes and incubated with 250 *μ*l of *α*-amylase solution, respectively, for 30 min at 25°C. In another set of tubes, *α*-amylase was incubated with 250 *μ*l of phosphate buffer (pH 6.9). Then, 250 *μ*l of starch solution at increasing concentrations (0.1–5.0 mg/ml) was added to the mixtures to start the reaction. The mixture was then incubated for 30 min at 25°C and then boiled for 5 min after addition of 500 *μ*l of DNS to stop the reaction. The amount of reducing sugars released was determined spectrophotometrically. This was followed by its conversion to reaction velocities. The type of *α*-amylase inhibition by the crude extract and its chloroform fraction was determined by Lineweaver–Burk plot (1/*v* versus 1/[S], where *v* is the reaction velocity and [S] is substrate concentration).

### 2.7. Isolation of *α*-Glucosidase from Rat's Small Intestine

The small intestine of male Sprague Dawley rat (180 g) was collected after sacrificing the animal. The intestine was thoroughly cleaned with normal saline, and epithelial layer (mucosal tissue) was collected by scraping the luminal surface firmly with a spatula. The mucosal scraping was homogenized in phosphate-buffered saline pH 7.4 containing 1 % triton X-100 and then centrifuged at 12000 rpm for 15 min. The supernatant fraction contained rat's small intestinal *α*-glucosidase. Butanol was added to the supernatant fraction 1 : 1 proportion and centrifuged at 15000 rpm for 15 min. The aqueous layer was dialyzed overnight against the same buffer. After dialysis, the concentrated enzyme was used as a crude *α*-glucosidase enzyme in the study [37].

### 2.8. Determination of *α*-Glucosidase Inhibition

The determination of *α*-glucosidase inhibition by *U. chamae* was carried out by using the modified method described by Kazeem et al. [36]. The isolated *α*-glucosidase (0.5 mg) from rat's small intestine was dissolved in 100 mM phosphate buffer pH 6.9. *p*-Nitrophenyl-*α*-D-glucopyranoside (pNPG) was used as the substrate. Plant extract and its fractions were used in the concentration ranging from 10–100 *μ*g/ml. Different concentrations of the crude extract or its fractions, chloroform, ethyl acetate, and ethanol, and *α*-glucosidase, were mixed with 320 *μ*l of 100 mM phosphate buffer pH 6.9 and incubated at 37°C for 10 minutes. Subsequently, the reaction was initiated by adding 50 *μ*l of 3 mM pNPG and incubated for 20 mins. The reaction was terminated by adding 3 ml of 50 mM sodium hydroxide, and the absorbance was read at 410 nm. The control samples were prepared without any plant extract or the fractions. The % inhibition was calculated according to the following formula:

(2) $$\begin{array}{c} \text{Inhibition}\;\;\left(\text{\%}\right)=\text{Abs}\;\;410\;\;\left(\text{control}\right)-\text{Abs}\;\;410\;\;\left(\text{extract}\right)\times \frac{100}{\text{Abs}\;\;410\;\;\left(\text{control}\right)}\text{.} \end{array}$$

The IC_50_ values were calculated by nonlinear regression analysis from the percentage inhibition. Acarbose was used as the control (the reference *α*-glucosidase inhibitor). All tests were performed in triplicate.

### 2.9. Determination of the Type of *α*-Glucosidase Inhibition

The determination of the type of *α*-glucosidase inhibition by *U. chamae* was assessed by using the crude extract and its ethanol fraction that had the lowest IC_50_. This was done according to the modified method described by Kazeem et al. [36]. The extract and ethanol fraction (50 *μ*l of 5 mg/ml) were incubated with 100 *μ*l of *α*-glucosidase solution, respectively, for 30 min at 25°C in two sets of tubes. In another set of tubes, *α*-glucosidase was incubated with 50 *μ*l of phosphate buffer (pH 6.9). The reaction was started by addition 50 *μ*l of pNPG at increasing concentrations (0.5–20 mM) to both sets of mixtures. The mixtures were then incubated for 10 min at 25°C, followed by addition of 500 *μ*l of sodium bicarbonate to stop the reaction. The quantity of reducing sugars released was determined spectrophotometrically. This was followed by its conversion to reaction velocities. The type of *α*-amylase inhibition by the crude extract and its chloroform fraction was determined by Lineweaver–Burk plot (1/*v* versus 1/[S], where *v* is the reaction velocity and [S] is the substrate concentration).

### 2.10. Analysis of Data

Analysis of data was carried out using GraphPad Prism 6 and SPSS version 22. GraphPad Prism was used for the diabetic study, and SPSS was used for the nonlinear regression analysis of *α*-amylase and glucosidase inhibitory activities. Nonlinear regression analysis was done with an R-square value of 0.9 and above and the IC_50_ values calculated from the regression analysis. The comparison of means of the groups was with one-way analysis of variance followed by Dunnett's post hoc test. The results were reported as mean ± SEM. The level of significance was set at *p* < 0.05.

## 3. Results

### 3.1. Effect of the Root Extract of *U. chamae* on Blood Glucose in Alloxan-Induced Diabetes Mellitus

On day one to day three, the blood glucose measurements of diabetic rats not treated and those treated with the root extract of *U. chamae*, glibenclamide, and pioglitazone were significantly (*p* < 0.05) elevated compared to the control (Table 1). However, on the 7th day to the end of the study, the 15th day, there was no significance difference in the blood glucose measurements of diabetic rats treated with the root extract of *U. chamae* compared to the control (Table 1). The rats treated with 100, 250, and 400 mg/kg of the root extract of *U. chamae* on the 7th day showed a marked blood glucose reduction of 72.14%, 78.75%, and 87.71%, respectively (Table 1). Conversely, the reference drugs glibenclamide and pioglitazone had a plasma glucose reduction of 63.10% and 30.46%, respectively. On the 15th day, the rats treated with 100, 250, and 400 mg/kg of the root extract of *U. chamae* showed a significant glucose reduction of 79.11%, 78.56%, and 88.11%, respectively, compared to the 74% and 55.07% glucose reduction of glibenclamide and pioglitazone, respectively (Table 1).

### 3.2. Effect of the Root Extract of *U. chamae* on Lipids in Alloxan-Induced Diabetes Mellitus

Effect of the root extract of *U. chamae* on plasma lipids is summarized in Table 2. There was no significant difference among the plasma LDL-cholesterol, TChol, and TG measurements of the diabetic rats treated with the root extract of *U. chamae* compared to the control. The HDL-cholesterol measurements of the diabetic rats treated with the root extract of *U. chamae* were significantly (*p* < 0.05) elevated compared to the control. While the LDL-cholesterol measurements of the diabetic rats not treated were significantly (*p* < 0.05) elevated compared to the control.

### 3.3. Effect of the Root Extract of *U. chamae* on Other Plasma Biochemical Parameters

Effect of the root extract of *U. chamae* on the other biochemical parameters is shown in Table 3. The root extract of *U. chamae* caused no significant alteration in the plasma creatinine, urea, protein, albumin, ALT, AST, and ALP measurements of the diabetic rats compared to the control. However, the plasma creatinine measurements were significantly (*p* < 0.05) elevated in diabetic rats not treated.

### 3.4. Effect of the Root Extract of *U. chamae* on Body Weight

The effect of the root extract of *U. chamae* on the body weight of the rats is summarized in Table 4. The root extract of *U. chamae* at doses of 100 and 400 mg/kg caused significant (*p* < 0.05) reduction in the weights of the rats from the 5th to 15th day of the study while 250 mg/kg of the extract caused no significant change in the weights of the rats except on the 11th day when the reduction became significant (*p* < 0.05) compared to the control. The weights of the diabetic rats that were not treated reduced significantly (*p* < 0.05) from the 7th to the 15th day of the study.

### 3.5. Effect of the Root Extract of *U. chamae* on the Blood Components

The effect of the root extract of *U. chamae* on the blood components is presented in Table 5. The root extract of *U. chamae* caused no significant alteration in white blood cell (WBC), red blood cell (RBC), hemoglobin concentration (Hgb), packed cell volume (PCV), mean corpuscular hemoglobin [38], mean corpuscular volume (MCV), mean corpuscular hemoglobin concentration (MCHC), and platelet (PLT) measurements of the diabetic rats compared to the control. However, glibenclamide caused a significant (*p* < 0.05) elevation in the WBC counts of the group of diabetic rats compared to the control.

### 3.6. Histological Findings

**Figure 1:**
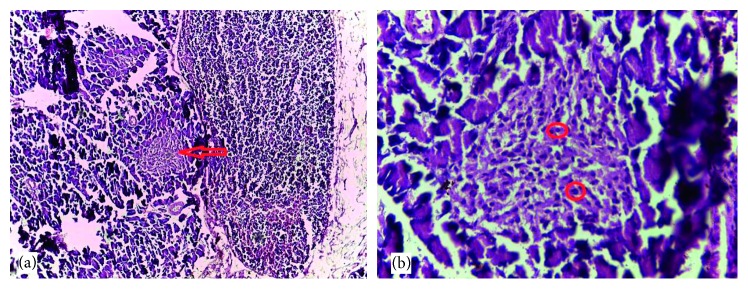
The tissue sections of control pancreas (H&E; (a) ×100; (b) ×400) show islets of Langerhans (a) and intact islet cells (b) and acinar cells with no remarkable alterations.

**Figure 2:**
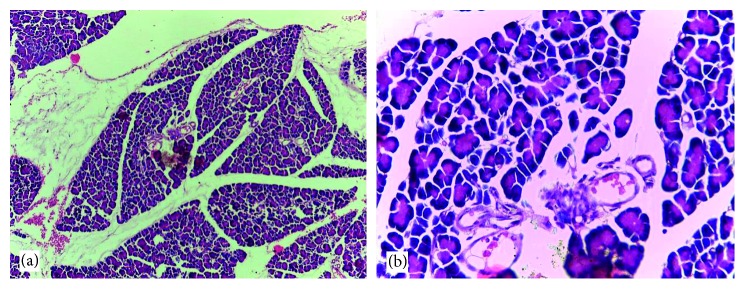
The tissue sections of the pancreas of diabetic rat treated with glibenclamide (71 *µ*g/kg) (H&E; (a) ×100; (b) ×400) showing no islet cells.

**Figure 3:**
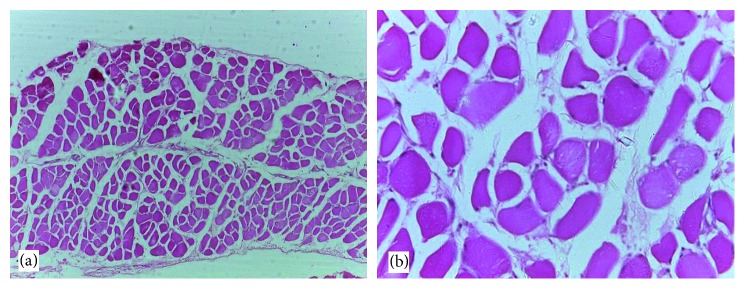
Tissue sections of the pancreas of diabetic rat treated with pioglitazone (429 *µ*g/kg) (H&E; (a) ×100; (b) ×400) showing no distinct islet cell regeneration in the tissue cross section.

**Figure 4:**
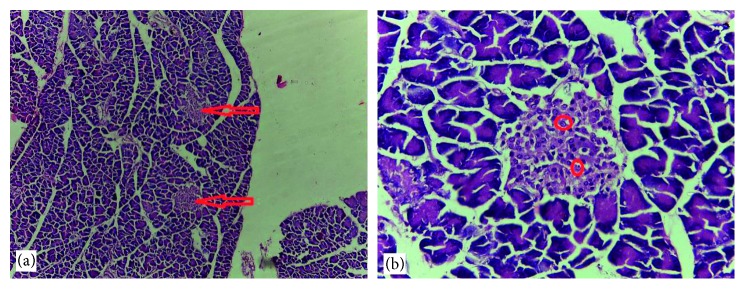
The islets of Langerhans (a), red arrow and regenerated islet cells (b), encircled in the tissue sections of the pancreas of diabetic rat treated with the root extract of *Uvaria chamae* (100 mg/kg) (H&E; (a) ×100; (b) ×400).

**Figure 5:**
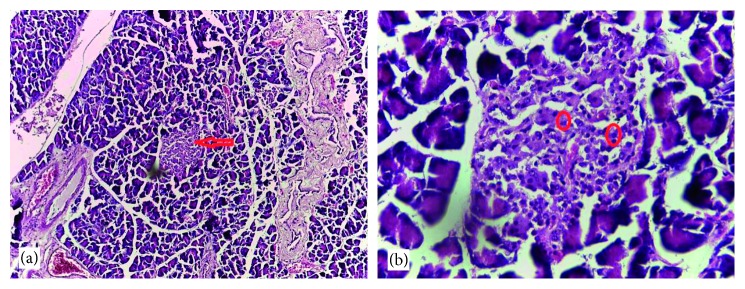
The islets of Langerhans (a), red arrow and regenerated islet cells (b), encircled in the tissue sections of the pancreas of diabetic rat treated with the root extract of *Uvaria chamae* (250 mg/kg) (H&E; (a) = ×100; (b) = ×400).

**Figure 6:**
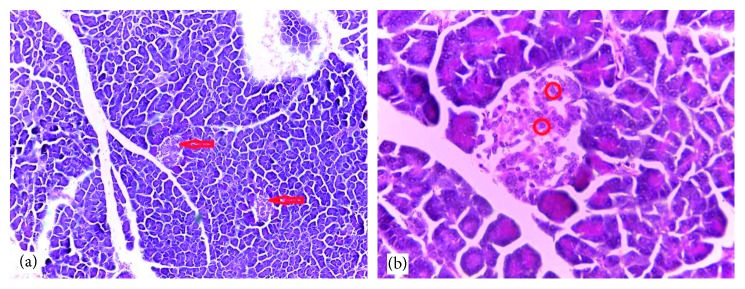
The islets of Langerhans (a), red arrow and regenerated islet cells (b), encircled in the tissue sections of the pancreas of diabetic rat treated with the root extract of *Uvaria chamae* (400 mg/kg) (H&E; (a) = ×100; (b) = ×400).

**Figure 7:**
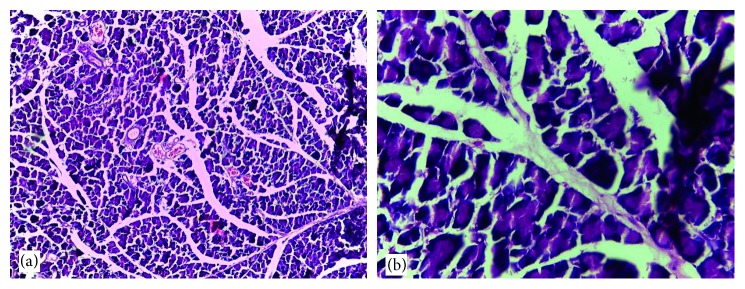
The distinct absence of islets of Langerhans in the tissue sections of the pancreas of diabetic rat not treated (H&E; (a) ×100; (b) ×400).

Normal cytoarchitectural features of the pancreas were observed in tissue sections of the normal control rats with intact and defined islets of Langerhans surrounded by acinar cells (Figure 1). The tissue sections of diabetic rats treated with glibenclamide (71 *µ*g/kg) (Figure 2) and pioglitazone (429 *µ*g/kg) (Figure 3) showed no visible islet cells, respectively. However, regenerated islet cells were seen (Figures 4–6) in the tissue sections of the pancreas of the diabetic rats treated with the root extract of *U. chamae* (100, 250, and 400 mg/kg, respectively) whereas, there was distinct absence of islets of Langerhans in the diabetic rats not treated (Figure 7).

### 3.7. Effect of the Root Extract of *U. chamae* on Insulin Secretion

**Figure 8:**
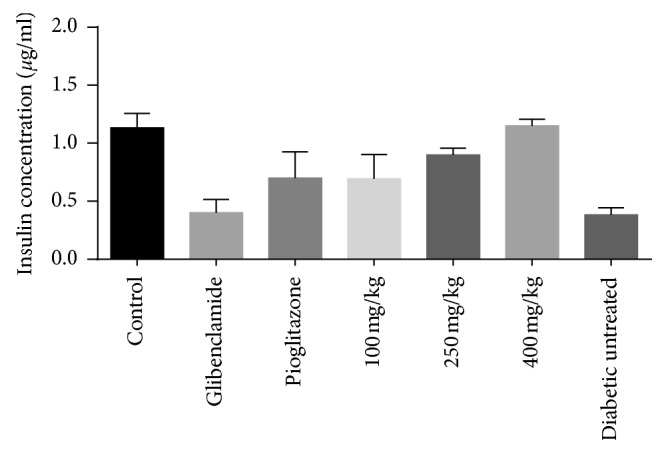
The increased insulin concentration from the serum of rats treated with the extract compared to the standard drugs (glibenclamide and pioglitazone).

The root extract of *U. chamae* caused a dose-dependent increase in insulin secretion with a marked increase in insulin concentration in the group of rats treated with 400 mg/kg of the extract compared to glibenclamide, pioglitazone, and diabetic untreated (Figure 8). However, there was no significant difference in insulin secretion in the diabetic rats treated with the extract compared to the control.

### 3.8. IC_50_ Values of *α*-Amylase and *α*-Glucosidase Inhibition

The summary of the calculated IC_50_ values from the nonlinear regression analysis is shown in Table 6. The chloroform fraction of *U. chamae* had the most effective inhibition of *α*-amylase with an IC_50_ value of −246.3 *μ*g/ml. However, the ethanolic fraction had the most effective inhibition of *α*-glucosidase with the IC_50_ value of −44.53 *μ*g/ml followed by the crude extract of *U. chamae* with the IC_50_ value of 15.29 *μ*g/ml.

### 3.9. *α*-Amylase Inhibition

**Figure 9:**
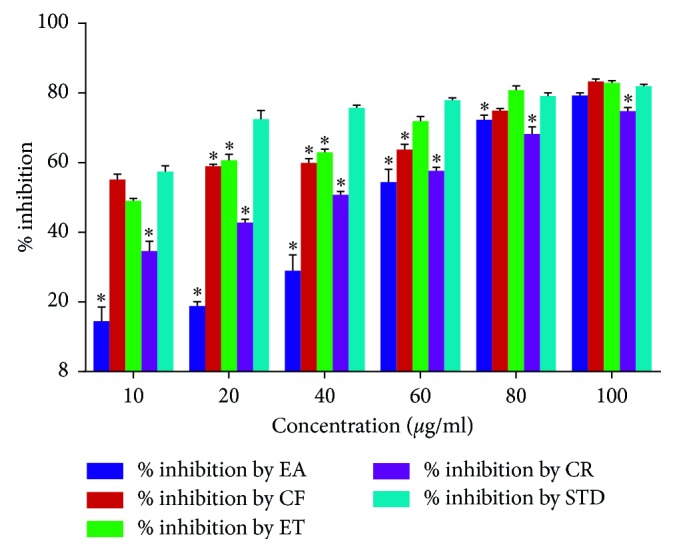
*α*-Amylase inhibition by *U. chamae* and its fractions. EA: ethyl acetate; CF: chloroform; ET: ethanol; CR: crude extract of *U. chamae*; STD: standard drug (acarbose). (*n*=3) ^*∗*^*p* < 0.05 vs standard (STD).

The summary of *α*-amylase inhibition by the crude extract of *U. chamae* and its fractions, ethyl acetate, chloroform, ethanol, and the reference drug (acarbose), is shown in Figure 9. The crude extract of *U. chamae* and its fractions caused a concentration-dependent inhibition of *α*-amylase. The chloroform and ethanolic fractions were more potent inhibitors of *α*-amylase.

### 3.10. The Type of *α*-Amylase Inhibition

**Figure 10:**
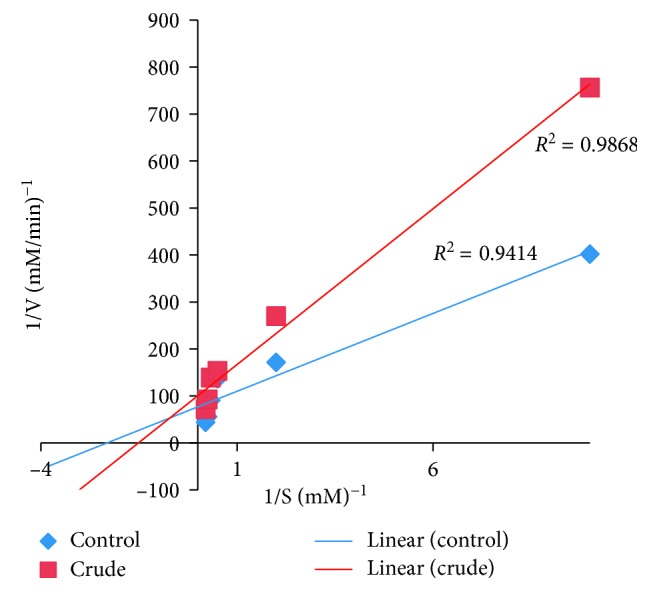
Noncompetitive *α*-amylase inhibition by *U. chamae*.

**Figure 11:**
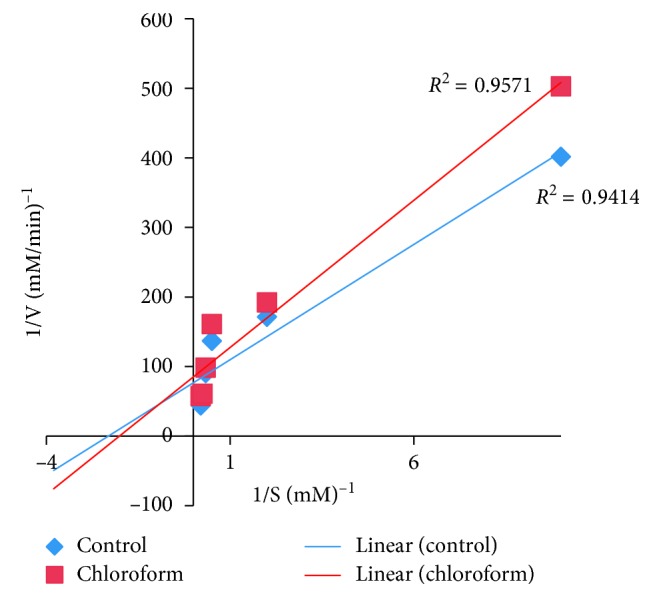
Noncompetitive *α*-amylase inhibition by chloroform fraction.

The type of *α*-amylase inhibition by the root extract of *U. chamae* and its chloroform fraction using Lineweaver–Burk plot showed that both the crude extract of *U. chamae* and its chloroform fraction exhibited a noncompetitive mode of inhibition (Figures 10 and 11), respectively.

### 3.11. *α-*Glucosidae Inhibition

**Figure 12:**
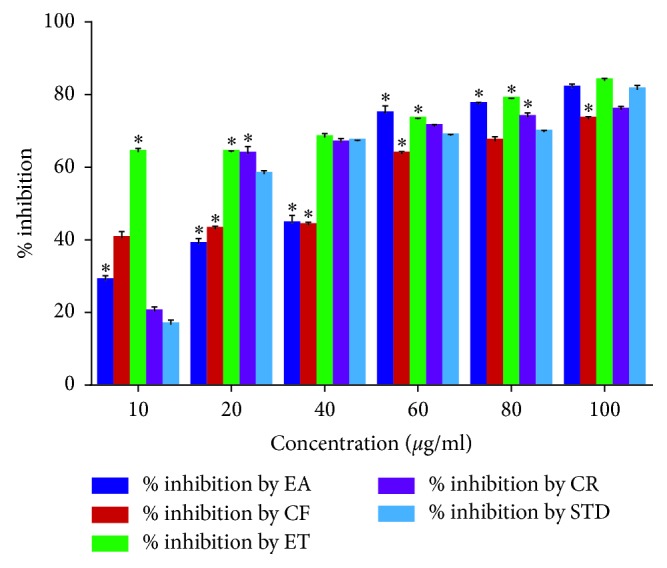
*α-*Glucosidae inhibition by *U. chamae* and its fractions. EA: ethyl acetate; CF: chloroform; ET: ethanol; CR: crude extract of *U. chamae*; STD: standard drug (acarbose). (*n*=3) ^*∗*^*p* < 0.05 vs standard (STD).

The summary of *α*-glucosidase inhibition by the root extract of *U. chamae* and its fractions, ethyl acetate, chloroform, ethanol, and the reference drug (acarbose), is presented in Figure 12. The crude extract of *U*. *chamae* caused a significant (*p* < 0.05) increase in the inhibition of *α*-glucosidase compared to the reference drug, acarbose. The ethanolic fraction was more potent than the other fractions.

### 3.12. Type of *α*-Glucosidase Inhibition

**Figure 13:**
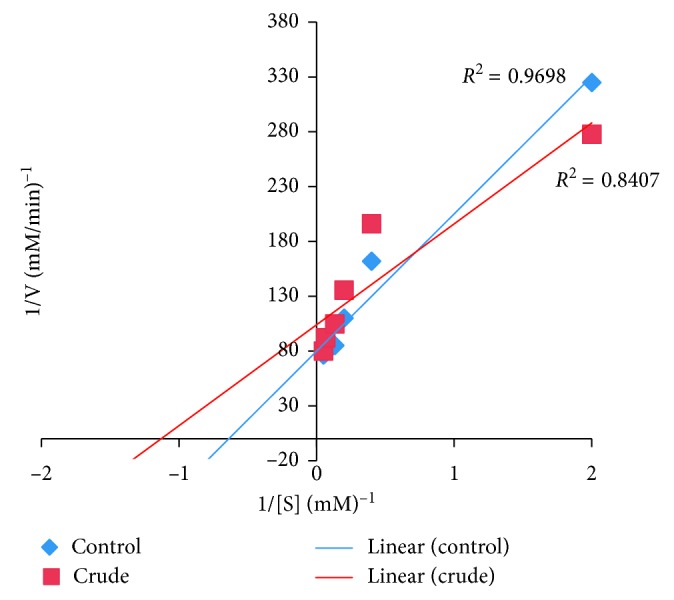
Competitive *α*-glucosidase inhibition by the crude extract.

**Figure 14:**
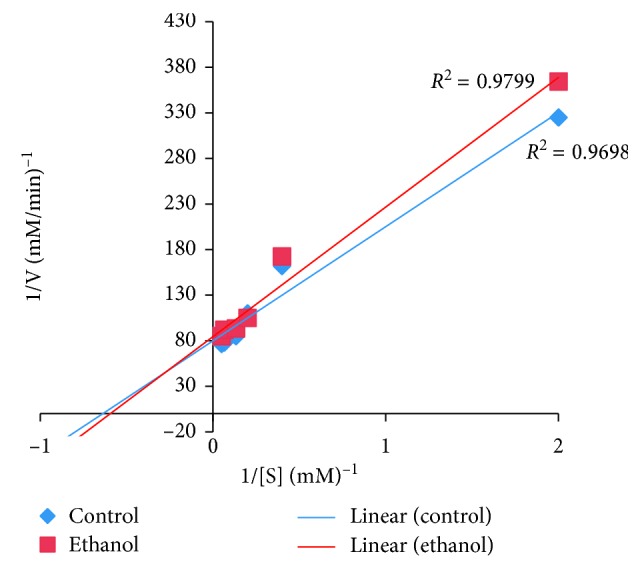
Noncompetitive *α*-glucosidase inhibition by the ethanolic fraction.

The type of *α*-glucosidase inhibition by the root extract of *U. chamae* and its ethanol fraction using Lineweaver–Burk plot showed that the crude extract and its ethanol fraction exhibited a competitive (Figure 13) and noncompetitive (Figure 14) type of inhibition, respectively.

## 4. Discussion

Diabetes mellitus was previously considered a disease of trivial importance to world health but is now regarded as a major public health challenge in the 21st century [39]. It is a disease characterized by chronic hyperglycemia in postprandial and fasting state with the risk of developing complications of the eyes, kidneys, peripheral nerves, heart, and blood vessels [40, 41]. These complications can be prevented by ensuring that the blood glucose measurements are within satisfactory limits [42]. Therefore, an important way of controlling diabetes mellitus is by the use of agents that reduce postprandial hyperglycemia by suppressing hydrolysis of carbohydrate [43]. The findings of this study revealed that the blood glucose levels of the diabetic rats treated with *U. chamae* were comparable to the normal control. Nevertheless, diabetic control was achieved on the 7th day with a marked glucose reduction of 72.14%, 78.75%, and 87.71% following the administration of 100, 250, and 400 mg/kg of the root extract of *U. chamae*, respectively. This glucose control was sustained till the end of the study. The reference drugs glibenclamide and pioglitazone had a plasma glucose reduction of 63.10% and 30.46%, respectively, on the 7th day. The antidiabetic activity of *U. chamae* may be from the inhibition of *α*-amylase and *α*-glucosidase. These are enzymes responsible for breaking *α*, 1, 4 bonds in complex carbohydrate [44]. The inhibition of these enzymes delays the breakdown of carbohydrate which leads to reduced blood glucose [45, 46]. The findings of this study indicated that *U. chamae* and its fractions ethyl acetate, chloroform, and ethanol caused *α*-amylase and *α*-glucosidase inhibition. The *α*-amylase inhibition of *U. chamae* and the chloroform fraction were noncompetitive. Noncompetitive inhibitors decrease turnover numbers rather than reduction of the proportion of enzyme molecules that are bound to the substrate [47]. Therefore, *U. chamae* as an *α*-amylase inhibitor decreased the conversion of polysaccharides and disaccharides to glucose in a given unit of time. The *α*-glucosidase inhibition of *U. chamae* and its ethanolic fraction were competitive and noncompetitive, respectively. A competitive inhibitor diminishes the rate of catalysis by reducing the proportion of enzyme molecules bound to a substrate [47]. Consequently, *U. chamae* as a competitive inhibitor of *α*-glucosidase may reduce absorption of glucose from the small intestine as glucose liberation from disaccharides is reduced. Therefore, the capacity of plant extracts to control the release and absorption of glucose is fast becoming an attractive therapeutic option in the treatment of diabetes mellitus [48]. The antidiabetic activity of *U. chamae* may also be from the presence of the secondary metabolites such as flavonoids, alkaloids, and tannins present in the plant [24]. Nevertheless, altered *β*-cell function and decreased *β*-cell mass may contribute to the defects in insulin release which is vital to the etiology of diabetes. These defects cause a progressive increase in glucose levels, with deterioration of glycemic control [6, 48, 49]. *U. chamae*'*s* ability to cause increased insulin secretion from the regenerated islet cells may also have been responsible for the antidiabetic activity of the plant. Studies have shown that replacement of pancreatic beta cells may restore blood glucose and holds the key to the cure of diabetes [50, 51]. Consequently, *U. chamae* by its ability to cause regeneration of the beta cells of the pancreas may possibly play a role as a potential therapeutic option for diabetes mellitus. Although this result may look inspiring, further studies are still required to measure quantitatively the beta cell mass. It should be stated however that dyslipidemia arising from diabetes mellitus is a risk factor for coronary heart disease [52]. The findings of this study showed that treatment of the diabetic rats with the root extract of *U. chamae* caused a significant elevation in the HDL-cholesterol with no significant alteration in the plasma LDL-cholesterol, TChol, and TG levels. The hyperlipidemia associated with diabetes mellitus is reduced by limited absorption of free fatty acids and free cholesterol following inhibition of pancreatic lipase and pancreatic cholesterol esterase [53, 54]. The high levels of plasma HDL-cholesterol prevent risk of developing cardiovascular disease [55, 56]. Nonetheless, long-term complications of diabetes emanate from sustained chronic hyperglycemia [57, 58]. This study revealed that the plasma creatinine levels of the diabetic rats untreated were significantly increased. This may be an indication of renal impairment in this group of rats [59]. However, the plasma creatinine and urea of diabetic rats treated with the root extract of *U. chamae* were normal suggesting that *U. chamae* is not nephrotoxic. Hepatotoxicity is marked by profound elevations in the plasma levels of liver enzymes (ALT, AST, and ALP) and at times reduced plasma total protein and albumin levels [60, 61]. These liver enzymes are used to screen for hepatobiliary disease and identify the liver damage from abuse of drugs or substances [62]. AST and ALT are also released into the plasma in large quantities whenever there is damage to the liver and heart [31]. Nevertheless, there were no significant alterations in the plasma AST, ALT, ALP, and other hepatic function parameters such as total protein and albumin in the diabetic rats that received the root extract of *U. chamae* indicating that *U. chamae* is not hepatotoxic and cardiotoxic. Studies have shown that intentional weight loss in diabetic patients may improve glycemic control and reduce cardiovascular disease and mortality [63, 64]. The root extract of *U. chamae* may be of value to diabetic patients that are obese as it causes weight loss. The hematological parameters provide vital information regarding the status of bone marrow activity and intravascular effects such as hemolysis and anemia [65]. The findings of this study revealed that there was no significant difference on the hematological parameters of the diabetic rats treated with the root extract of *U. chamae* suggesting that *U. chamae* did not cause anemia and thrombosis nor suppressed the immune system. However, an increase in WBC count caused by glibenclamide is suggestive of boost immunity.

## 5. Conclusion

The study demonstrated the antidiabetic effects of *U. chamae* which may be through *α*-amylase and *α*-glucosidase inhibition and increased insulin secretion from the regenerated pancreatic beta cells. The plant also showed a cardioprotective effect via an increase in HDL-cholesterol levels.

## Figures and Tables

**Table 1:** Effect of the root extract of *U. chamae* on blood glucose (mg/dl) in alloxan-induced diabetes mellitus and percentage reduction in blood glucose (%).

Days	Control (2% acacia)	Glibenclamide (71 *µ*g/kg)	Pioglitazone (429 *µ*g/kg)	UC (100 mg/kg)	UC (250 mg/kg)	UC (400 mg/kg)	Diabetic untreated
0	86.75 ± 2.36	76.00 ± 3.22	83.33 ± 9.20	88.33 ± 3.76	71.33 ± 3.76	77.67 ± 4.26	65.33 ± 5.90
1	91.75 ± 1.93	357.7 ± 28.30^*∗*^	352.3 ± 9.40^*∗*^	359.0 ± 16.10^*∗*^	337.3 ± 6.70^*∗*^	501.7 ± 56.30^*∗*^	264.0 ± 5.80^*∗*^
3	82.00 ± 3.20	222.3 ± 51.40^*∗*^ (37.85)	239.0 ± 38.40^*∗*^ (32.16)	284.7 ± 22.40^*∗*^ (20.70)	270.7 ± 18.8^*∗*^ (19.75)	280.3 ± 8.30^*∗*^ (44.13)	272.0 ± 2.00^*∗*^ (−3.03)
5	84.00 ± 3.00	189.0 ± 48.80 (47.16)	173.7 ± 35.80 (50.70)	198.7 ± 64.30 (44.65)	94.0 ± 7.50 (72.13)	157.0 ± 61.10 (68.71)	282.3 ± 1.50^*∗*^ (−6.93)
7	78.25 ± 4.27	132.0 ± 16.80 (63.10)	245.0 ± 59.81^*∗*^ (30.46)	100.0 ± 11.68 (72.14)	71.67 ± 9.17 (78.75)	61.67 ± 1.86 (87.71)	288.7 ± 0.88^*∗*^ (−9.36)
9	90.25 ± 3.10	117.0 ± 11.90 (67.29)	275.3 ± 48.40^*∗*^ (21.86)	91.67 ± 13.60 (74.47)	70.33 ± 8.70 (79.15)	58.33 ± 6.40 (88.37)	297.7 ± 1.20^*∗*^ (−12.77)
11	84.75 ± 2.50	109.3 ± 10.30 (69.44)	202.3 ± 70.50 (42.58)	90.33 ± 9.20 (74.84)	73.0 ± 4.00 (78.36)	50.33 ± 7.40 (89.97)	305.3 ± 4.80^*∗*^ (−15.64)
13	83.50 ± 4.90	100.0 ± 7.50 (72.04)	145.7 ± 77.70 (58.64)	79.0 ± 10.70 (77.99)	68.33 ± 2.00 (79.74)	55.0 ± 7.60 (89.04)	312.7 ± 9.20^*∗*^ (−18.45)
15	79.75 ± 3.50	93.0 ± 5.60 (74.00)	158.3 ± 59.30 (55.07)	75.0 ± 10.00 (79.11)	72.33 ± 4.30 (78.56)	59.67 ± 6.40 (88.11)	318.7 ± 11.30^*∗*^ (−20.72)

^*∗*^Significant difference (*p* < 0.05; *n*=5) between the mean ± SEM of test groups vs. control. UC: *Uvaria chamae*.

**Table 2:** Effect of the root extract of *U. chamae* on lipids (mg/dl) in alloxan-induced diabetes mellitus.

Parameters	Control (2% acacia)	Glibenclamide (71 *µ*g/kg)	Pioglitazone (429 *µ*g/kg)	UC (100 mg/kg)	UC (250 mg/kg)	UC (400 mg/kg)	Diabetic untreated
TChol	151.3 ± 4.67	168.7 ± 17.4	172.3 ± 13.0	176.0 ± 5.0	163.0 ± 13.8	163.0 ± 1.8	179.3 ± 11.1
TG	51.33 ± 9.40	53.3 ± 6.8	57.7 ± 7.80	55.3 ± 5.90	43.3 ± 3.80	46.0 ± 5.03	65.7 ± 0.33
HDL	35.33 ± 0.88	47.33 ± 4.97	41.67 ± 4.97	44.67 ± 2.4^*∗*^	50.0 ± 2.88^*∗*^	57.67 ± 1.5^*∗*^	33.67 ± 3.18
LDL	105.7 ± 0.60	110.7 ± 1.3	119.1 ± 1.03	120.3 ± 0.52	104.3 ± 0.12	96.1 ± 0.30	132.2 ± 0.30^*∗*^

^*∗*^Significant difference (*p* < 0.05; *n*=5) between the mean ± SEM of test groups vs. control. UC: *Uvaria chamae*.

**Table 3:** Effect of the root extract of *U. chamae* on other plasma biochemical parameters.

Parameters	Control (2% acacia)	Glibenclamide (71 *µ*g/kg)	Pioglitazone (429 *µ*g/kg)	UC (100 mg/kg)	UC (250 mg/kg)	UC (400 mg/kg)	Diabetic untreated
AST (U/L)	21.67 ± 4.26	30.33 ± 4.10	27.67 ± 4.63	23.0 ± 1.53	27.67 ± 2.03	26.67 ± 0.88	30.0 ± 1.16
ALT (U/L)	15.67 ± 1.33	26.67 ± 3.18	18.33 ± 4.49	19.33 ± 6.00	22.67 ± 3.18	24.33 ± 3.38	17.33 ± 2.03
ALP (U/L)	30.67 ± 5.70	30.67 ± 3.53	30.67 ± 4.18	21.00 ± 2.08	28.33 ± 6.49	28.00 ± 6.55	34.0 ± 6.66
Creatinine (mg/dl)	0.83 ± 0.03	1.00 ± 0.10	0.80 ± 0.10	1.03 ± 0.09	0.83 ± 0.03	0.80 ± 0.06	1.13 ± 0.03^*∗*^
Urea (mg/dl)	38.00 ± 7.02	39.67 ± 7.05	33.67 ± 4.81	32.00 ± 1.16	26.67 ± 3.48	34.0 ± 3.22	44.67 ± 5.36
Protein (g/dl)	6.90 ± 0.62	5.97 ± 0.54	6.73 ± 0.38	6.47 ± 0.58	6.13 ± 0.59	6.70 ± 0.38	6.97 ± 0.09
ALB (mg/dl)	3.40 ± 0.35	2.83 ± 0.38	3.73 ± 0.32	3.23 ± 0.30	3.03 ± 0.20	3.53 ± 0.29	3.20 ± 0.20

^*∗*^Significant difference (*p* < 0.05; *n*=5) between the mean ± SEM of test groups vs. control. UC: *Uvaria chamae*.

**Table 4:** Effect of the root extract of *U. chamae* on body weight (g).

Days	Control (2% acacia)	Glibenclamide (71 *µ*g/kg)	Pioglitazone (429 *µ*/kg)	UC (100 mg/kg)	UC (250 mg/kg)	UC (400 mg/kg)	Diabetic untreated
1	146.0 ± 0.57	124.0 ± 10.69	153.0 ± 4.04	139.3 ± 9.35	156.0 ± 6.81	124.7 ± 3.71	131.3 ± 4.18
3	144.7 ± 0.33	115.7 ± 4.80^*∗*^	152.0 ± 5.29	135.3 ± 7.42	154.3 ± 7.54	123.3 ± 3.33	130.0 ± 5.29
5	140.7 ± 2.33	119.7 ± 4.91^*∗*^	129.7 ± 1.86	111.7 ± 7.88^*∗*^	143.7 ± 7.3	119.0 ± 2.65^*∗*^	125.0 ± 2.89
7	144.0 ± 2.08	122.7 ± 5.90^*∗*^	131.0 ± 2.65	122.7 ± 3.71^*∗*^	143.7 ± 5.78	117.3 ± 1.76^*∗*^	119.3 ± 4.26^*∗*^
9	144.7 ± 2.67	118.3 ± 9.28^*∗*^	127.3 ± 2.91	113.7 ± 5.81^*∗*^	132.3 ± 6.69	114.7 ± 2.91^*∗*^	107.3 ± 4.06^*∗*^
11	145.0 ± 2.65	131.3 ± 7.69	127.3 ± 2.90	114.3 ± 3.48^*∗*^	109.7 ± 4.84^*∗*^	110.0 ± 2.65^*∗*^	115.3 ± 4.33^*∗*^
13	149.7 ± 3.18	132.0 ± 3.61^*∗*^	141.3 ± 2.60	112.3 ± 1.45^*∗*^	136.0 ± 5.69	107.0 ± 5.57^*∗*^	109.3 ± 3.71^*∗*^
15	155.0 ± 3.51	133.3 ± 1.76^*∗*^	141.7 ± 2.85	103.7 ± 2.33^*∗*^	137.3 ± 1.20	106.3 ± 8.95^*∗*^	116.0 ± 5.51^*∗*^

^*∗*^Significant difference (*p* < 0.05; *n*=5) between the mean ± SEM of test groups vs. control. UC: *Uvaria chamae*.

**Table 5:** The effect of the root extract of *U. chamae* on the blood components.

Parameters	Control (2% acacia)	Glibenclamide (71 *µ*g/kg)	Pioglitazone (429 *µ*/kg)	UC (100 mg/kg)	UC (250 mg/kg)	UC (400 mg/kg)	Diabetic untreated
WBC (×10^9^/L)	6.9 ± 0.47	11.9 ± 1.22^*∗*^	9.6 ± 0.70	5.2 ± 0.36	6.4 ± 0.56	6.3 ± 0.64	9.17 ± 0.61
RBC (×10^12^/L)	5.8 ± 0.30	5.2 ± 0.26	5.8 ± 0.50	5.5 ± 0.90	6.0 ± 0.48	5.8 ± 0.31	6.3 ± 0.62
Hgb (g/dl)	12.0 ± 0.6	10.0 ± 0.43	10.3 ± 0.79	10.0 ± 1.64	10.8 ± 0.8	10.0 ± 0.12	12.4 ± 1.69
PCV (%)	40.3 ± 3.38	29.9 ± 0.67	30.23 ± 2.10	32.0 ± 5.74	32.9 ± 2.64	32.8 ± 1.48	38.10 ± 5.29
MCV (fL)	70.5 ± 7.0	54.6 ± 2.24	56.0 ± 2.52	58.4 ± 2.17	55.3 ± 2.95	59.3 ± 4.33	60.2 ± 3.23
MCH (pg)	20.9 ± 1.29	19.3 ± 0.70	17.9 ± 0.23	18.4 ± 0.43	18.0 ± 0.94	18.5 ± 0.67	19.5 ± 0.92
MCHC (g/dl)	30.0 ± 1.30	33.3 ± 0.67	34.0 ± 0.35	31.3 ± 0.64	32.8 ± 0.03	32.2 ± 1.58	32.5 ± 0.30
PLT (×10^9^/L)	918.7 ± 5.90	944.0 ± 25.74	958.0 ± 34.70	971.3 ± 8.99	967.7 ± 22.60	929.0 ± 17.04	955.0 ± 26.63

^*∗*^Significant difference (*p* < 0.05; *n*=5) between the mean ± SEM of test groups vs. control. UC: *Uvaria chamae*.

**Table 6:** IC_50_ values of *α*-amylase and *α*-glucosidase inhibition.

Extract/fractions	IC_50_ (*μ*g/ml)	
	*α*-Amylase	*α*-Glucosidase
*Uvaria chamae*	40.64	15.29
Ethyl acetate	57.52	34.38
Chloroform	−246.3	43.99
Ethanol	10.96	−44.53
Acarbose	3.12	15.89

## Data Availability

The data used to support the findings of this study are included within the article.

## Abbreviations

DM: Diabetes mellitus
FBG: Fasting blood glucose
EDTA: Ethylenediaminetetraacetate
TChol: Total cholesterol
TG: Triglyceride
HDL: High-density lipoprotein
LDL: Low-density lipoprotein
AST: Aspartate aminotransferase
ALT: Alanine aminotransferase
ALP: Alkaline phosphatase
H&E: Haematoxylin and Eosin
DNS: Dinitrosalicylic acid
Abs: Absorbance
IC_50_: Concentrations of extracts or fractions resulting in 50% inhibition of enzyme activity, v: reaction velocity
[S]: Substrate concentration
SPSS: Statistical Package for the Social Sciences
ANOVA: Analysis of variance
SEM: Standard error of mean
WBC: White blood cell
RBC: Red blood cell
Hgb: Hemoglobin
PCV: Packed cell volume
MCH: Mean cell hemoglobin
MCV: Mean corpuscular volume
MCHC: Mean corpuscular hemoglobin concentration
PLT: Platelet
EA: Ethyl acetate
CF: Chloroform
ET: Ethanol
CR: Crude extract
STD: Standard (acarbose).

## Supplementary Materials

Supplementary Materials In the supplementary material, the nonlinear regression analysis was used to calculate the (IC_50_: concentrations of extracts or fractions resulting in 50% inhibition of enzyme activity) IC_50_ values of the fractions and the crude extract of *U. chamae* against the enzymes (*α*-amylase and *α*-glucosidase). In the study, the lower the IC_50_ values, the more potent the fraction is in inhibiting the enzymes. Therefore, we are more likely to focus on evaluating the fractions with the lowest IC_50_ in future investigation. Details of results showing the different IC_50_ values are sufficiently indicated in the main manuscript.

## References

[1] Tabish S. A. (2007). Is diabetes becoming the biggest epidemic of the twenty-first century?. *International Journal of Health Sciences*.

[2] World Health Organization (2014). *Global Health estimates 2013: Deaths by Cause, Age, Sex and Country, 2000-2012*.

[3] Andrade F. C. D. (2009). Measuring the impact of diabetes on life expectancy and disability-free life expectancy among older adults in Mexico. *Journals of Gerontology Series B: Psychological Sciences and Social Sciences*.

[4] Alberti K. G. M. M., Zimmet P. Z., WHO Consultation (1998). Definition, diagnosis and classification of diabetes mellitus and its complications. Part 1: diagnosis and classification of diabetes mellitus. Provisional report of a WHO Consultation. *Diabetic Medicine*.

[5] American Diabetes Association (2010). Diagnosis and classification of diabetes mellitus. *Diabetes Care*.

[6] Cerf M. E. (2013). Beta cell dysfunction and insulin resistance. *Frontiers in Endocrinology*.

[7] Deshpande A. D., Harris-Hayes M., Schootman M. (2008). Epidemiology of diabetes and diabetes-related complications. *Physical Therapy*.

[8] Ray J. A., Valentine W. J., Secnik K. (2005). Review of the cost of diabetes complications in Australia, Canada, France, Germany, Italy and Spain. *Current Medical Research and Opinion*.

[9] Scully T. (2012). Diabetes in numbers. *Nature*.

[10] Matsumoto S., Noguchi H., Hatanaka N. (2009). Estimation of donor usability for islet transplantation in the United States with the Kyoto islet isolation method. *Cell Transplantation*.

[11] Abdel Aziz M. T., El-Asmar M. F., Rezq A. M. (2013). The effect of a novel curcumin derivative on pancreatic islet regeneration in experimental type-1 diabetes in rats (long term study). *Diabetology and Metabolic Syndrome*.

[12] Xu X., Wang G., Zhou T., Chen L., Chen J., Shen X. (2014). Novel approaches to drug discovery for the treatment of type 2 diabetes. *Expert Opinion on Drug Discovery*.

[13] Maedler K., Carr R. D., Bosco D., Zuellig R. A., Berney T., Donath M. Y. (2005). Sulfonylurea induced β-cell apoptosis in cultured human islets. *Journal of Clinical Endocrinology and Metabolism*.

[14] Del Guerra S., Marselli L., Lupi R. (2005). Effects of prolonged in vitro exposure to sulphonylureas on the function and survival of human islets. *Journal of Diabetes and its Complications*.

[15] Tahrani A. A., Bailey C. J., Del Prato S., Barnett A. H. (2011). Management of type 2 diabetes: new and future developments in treatment. *The Lancet*.

[16] Meier J. J. (2008). Beta cell mass in diabetes: a realistic therapeutic target?. *Diabetologia*.

[17] Risbud M. V., Bhonde R. R. (2002). Models of pancreatic regeneration in diabetes. *Diabetes Research and Clinical Practice*.

[18] Jun H.-S. (2008). Regeneration of pancreatic beta cells. *Frontiers in Bioscience*.

[19] Xiu L.-M., Miura A. B., Yamamoto K. (2001). Pancreatic islet regeneration by ephedrine in mice with streptozotocin-induced diabetes. *American Journal of Chinese Medicine*.

[20] Mahomoodally M. F. (2013). Traditional medicines in Africa: an appraisal of ten potent african medicinal plants. *Evidence-Based Complementary and Alternative Medicine*.

[21] Okwu D. E., Iroabuchi F. (2009). Phytochemical composition and biological activities of *Uvaria chamae* and clerodendoron splendens. *E-Journal of Chemistry*.

[22] Oluremi B., Osungunna M., Omafuma O. (2010). Comparative assessment of antibacterial activity of *Uvaria chamae* parts. *African Journal of Microbiology Research*.

[23] Emordi J. E., Agbaje E. O., Oreagba I. A., Iribhogbe O. I. (2016). Antidiabetic and hypolipidemic activities of hydroethanolic root extract of *Uvaria chamae* in streptozotocin induced diabetic albino rats. *BMC Complementary and Alternative Medicine*.

[24] Emeka E. J., Oluwatoyin A. E., Adekunle O. I., Ignis I. O. (2015). Preliminary phytochemical screening and evaluation of hypoglycemic properties of the root extract of *Uvaria chamae*. *Bangladesh Journal of Pharmacology*.

[25] Okokon J. E., Ita B. N., Udokpoh A. E. (2006). The in-vivo antimalarial activities of *Uvaria chamae* and *Hippocratea africana*. *Annals of Tropical Medicine and Parasitology*.

[26] Okwuosa O., Chukwura E., Chukwuma G. (2012). Phytochemical and antifungal activities of *Uvaria chamae* leaves and roots, *Spondias mombin* leaves and bark and *Combretum racemosum* leaves. *African Journal of Medicine and Medical Sciences*.

[27] Adelodun V. O., Elusiyan C. A., Olorunmola F. O. (2013). Evaluation of antitrypanosomal and anti inflammatory activities of selected Nigerian medicinal plants in mice. *African Journal of Traditional, Complementary and Alternative Medicines*.

[28] Council N. R. (2010). *Guide for the Care and Use of Laboratory Animals*.

[29] Fard M. H., Naseh G., Lotfi N., Hosseini S. M., Hosseini M. (2015). Effects of aqueous extract of turnip leaf (*Brassica rapa*) in alloxan-induced diabetic rats. *Avicenna Journal of Phytomedicine*.

[30] Migliori G. B., Pontali E., Sotgiu G. (2017). Combined use of Delamanid and Bedaquiline to treat multidrug-resistant and extensively drug-resistant tuberculosis: a systematic review. *International Journal of Molecular Sciences*.

[31] Crook M. (2006). *Clinical Chemistry & Metabolic Medicine*.

[32] Bagheri H., Michel F., Lapeyre-Mestre M. (2001). Detection and incidence of drug-induced liver injuries in hospital: a prospective analysis from laboratory signals. *British Journal of Clinical Pharmacology*.

[33] Hørder M., Elser R., Gerhardt W., Mathieu M., Sampson E. (1991). Approved recommendation of IFCC methods for the measurement of catalytic concentration of enzymes. VII: IFCC method for creatine kinase (ATP: creatine N-phosphotransferase EC 2.7. 3.2). *European Journal of Clinical Chemistry and Clinical Biochemistry*.

[34] Ali Hussain H. E. M. (2002). Hypoglycemic, hypolipidemic and antioxidant properties of combination of Curcumin from Curcuma longa, Linn, and partially purified product from *Abroma augusta*, Linn. in streptozotocin induced diabetes. *Indian Journal of Clinical Biochemistry*.

[35] Grizzle W. E., Fredenburgh J. L., Myers R. B. (2008). *Fixation of Tissues, Theory and Practice of Histological Techniques*.

[36] Kazeem M. I., Ogungbe S. M., Saibu G. M., Aboyade O. M. (2014). In vitro study on the hypoglycemic potential of *Nicotiana tabacum* leaf extracts. *Bangladesh Journal of Pharmacology*.

[37] Nagmoti D. M., Juvekar A. R. (2013). In vitro inhibitory effects of *Pithecellobium dulce* (Roxb.) Benth. seeds on intestinal *α*-glucosidase and pancreatic *α*-amylase. *Journal of Biochemical Technology*.

[38] Ayisi J. G., van’t Hoog A. H., Agaya J. A. (2011). Care seeking and attitudes towards treatment compliance by newly enrolled tuberculosis patients in the district treatment programme in rural western Kenya: a qualitative study. *BMC Public Health*.

[39] Masur K., Thévenod F., Zänker K. S. (2008). *Diabetes and Cancer: Epidemiological Evidence and Molecular Links*.

[40] Athanasakis K., Ollandezos M., Angeli A., Gregoriou A., Geitona M., Kyriopoulos J. (2010). Estimating the direct cost of type 2 diabetes in Greece: the effects of blood glucose regulation on patient cost. *Diabetic Medicine*.

[41] Olaokun O. O., McGaw L. J., van Rensburg I. J., Eloff J. N., Naidoo V. (2016). Antidiabetic activity of the ethyl acetate fraction of *Ficus lutea* (Moraceae) leaf extract: comparison of an in vitro assay with an in vivo obese mouse model. *BMC Complementary and Alternative Medicine*.

[42] Park J. M., Bong H. Y., Jeong H. I., Kim Y. K., Kim J. Y., Kwon O. (2009). Postprandial hypoglycemic effect of mulberry leaf in Goto-Kakizaki rats and counterpart control Wistar rats. *Nutrition Research and Practice*.

[43] Mahomoodally M. F., Subratty A. H., Gurib-Fakim A., Choudhary M. I., Khan S. N. (2012). Traditional medicinal herbs and food plants have the potential to inhibit key carbohydrate hydrolyzing Enzymes in vitro and reduce postprandial blood glucose peaks in vivo. *Scientific World Journal*.

[44] Picot C. M. N., Subratty A. H., Mahomoodally M. F. (2014). Inhibitory potential of five traditionally used native antidiabetic medicinal plants on *α*-amylase, *α*-glucosidase, glucose entrapment, and amylolysis Kinetics in vitro. *Advances in Pharmacological Sciences*.

[45] Prabhu A. S., Ananthan G. (2014). Alpha-amylase inhibitory activities of ascidians in the treatment of diabetes mellitus. *Bangladesh Journal of Pharmacology*.

[46] Jo S.-H., Cho C.-Y., Lee J.-Y., Ha K.-S., Kwon Y.-I., Apostolidis E. (2016). In vitro and in vivo reduction of post-prandial blood glucose levels by ethyl alcohol and water *Zingiber mioga* extracts through the inhibition of carbohydrate hydrolyzing enzymes. *BMC Complementary and Alternative Medicine*.

[47] Berg J. M., Tymoczko J. (2002). *Stryer: Biochemistry*.

[48] Thilagam E., Parimaladevi B., Kumarappan C., Mandal S. C. (2013). *α*-Glucosidase and *α*-amylase inhibitory activity of *Senna surattensis*. *Journal of Acupuncture and Meridian Studies*.

[49] Stoffers D. A. (2004). The development of beta-cell mass: recent progress and potential role of GLP-1. *Hormone and Metabolic Research*.

[50] Wang S., Hecksher-Sorensen J., Xu Y. (2008). Myt1 and Ngn3 form a feed-forward expression loop to promote endocrine islet cell differentiation. *Developmental Biology*.

[51] Feng Z. C., Li J., Turco B. A., Riopel M., Yee S. P., Wang R. (2012). Critical role of c-Kit in beta cell function: increased insulin secretion and protection against diabetes in a mouse model. *Diabetologia*.

[52] Thirumalaisamy B., Prabhakaran S. G., Marimuthu K., Chatterjee T. K. (2013). Antihyperlipidemic activity of the ethyl-acetate fraction of stereospermum suaveolens in streptozotocin-induced diabetic rats. *Journal of Pharmacopuncture*.

[53] Heidrich J. E., Contos L. M., Hunsaker L. A., Deck L. M., Vander Jagt D. L. (2004). Inhibition of pancreatic cholesterol esterase reduces cholesterol absorption in the hamster. *BMC Pharmacology*.

[54] Birari R. B., Bhutani K. K. (2007). Pancreatic lipase inhibitors from natural sources: unexplored potential. *Drug Discovery Today*.

[55] Ali K. M., Wonnerth A., Huber K., Wojta J. (2012). Cardiovascular disease risk reduction by raising HDL cholesterol - current therapies and future opportunities. *British Journal of Pharmacology*.

[56] Chang H., Wang Q., Shi T. (2016). Effect of DanQi Pill on PPAR*α*, lipid disorders and arachidonic acid pathway in rat model of coronary heart disease. *BMC Complementary and Alternative Medicine*.

[57] Adisakwattana S., Thilavech T., Chusak C. (2014). Mesona Chinensis Benth extract prevents AGE formation and protein oxidation against fructose-induced protein glycation in vitro. *BMC Complementary and Alternative Medicine*.

[58] Perera H. K. I., Premadasa W. K. V. K., Poongunran J. (2015). *α*-glucosidase and glycation inhibitory effects of costus speciosus leaves. *BMC Complementary and Alternative Medicine*.

[59] William M., Stephen B. (2008). *Clinical Chemistry*.

[60] Caisey J. D., King D. J. (1980). Clinical chemical values for some common laboratory animals. *Clinical Chemistry*.

[61] Woodman D. D. (1996). Assessment of hepatotoxicity. *Animal Clinical Chemistry*.

[62] Senior J. R. (2009). Monitoring for hepatotoxicity: what is the predictive value of liver “function” tests?. *Clinical Pharmacology and Therapeutics*.

[63] Williamson D. F., Thompson T. J., Thun M., Flanders D., Pamuk E., Byers T. (2000). Intentional weight loss and mortality among overweight individuals with diabetes. *Diabetes Care*.

[64] Hollander P. (2007). Anti-diabetes and anti-obesity medications: effects on weight in people with diabetes. *Diabetes Spectrum*.

[65] Akindele A. J., Adeneye A. A., Salau O. S., Sofidiya M. O., Benebo A. S. (2014). Dose and time-dependent sub-chronic toxicity study of hydroethanolic leaf extract of *Flabellaria paniculata* Cav. (Malpighiaceae) in rodents. *Frontiers in Pharmacology*.

