# Supplementary material for: Antidiabetic Effects of the Ethanolic Root Extract of Uvaria chamae P. Beauv (Annonaceae) in Alloxan-Induced Diabetic Rats: A Potential Alternative Treatment for Diabetes Mellitus
Source: Adv Pharmacol Sci. 2018 Nov 8;2018:1314941. doi: 10.1155/2018/1314941 (PMC6250042; doi:10.1155/2018/1314941)
Supplement: Supplementary Materials — In the supplementary material, the nonlinear regression analysis was used to calculate the (IC50: concentrations of extracts or fractions resulting in 50% inhibition of enzyme activity) IC50 values of the fractions and the crude extract of U. chamae against the enzymes (α-amylase and α-glucosidase). In the study, the lower the IC50 values, the more potent the fraction is in inhibiting the enzymes. Therefore, we are more likely to focus on evaluating the fractions with the lowest IC50 in future investigation. Details of results showing the different IC50 values are sufficiently indicated in the main manuscript. [file 1314941.f1.doc]

**REGRESSION FOR THE DATA**

Model 1: Nonlinear regression

Data 1: IC_50_ of Ethyl acetate fraction against **α-amylase**

| Concentration (μg/ml) | 10 | 20 | 40 | 60 | 80 | 100 |
| --- | --- | --- | --- | --- | --- | --- |
| % Inhibition | 14.45 | 18.82 | 28.9 | 54.37 | 72.24 | 79.28 |

Model equation


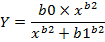


| **Parameter Estimates** | | | | | | | | |
| --- | --- | --- | --- | --- | --- | --- | --- | --- |
| Parameter | Estimate | | Std. Error | | 95% Confidence Interval | | | |
|  |  |  |  |  | Lower Bound | | Upper Bound | |
| b0 | 5311.375 | | 296212.160 | | -937367.918 | | 947990.669 | |
| b1 | 9379.136 | | 602470.767 | | -1907951.729 | | 1926710.001 | |
| b2 | .914 | | .482 | | -.619 | | 2.448 | |
| **ANOVA^a^** | | | | | | | |  |
| Source | | Sum of Squares | | Df | | Mean Squares | |  |
| Regression | | 15750.783 | | 3 | | 5250.261 | |  |
| Residual | | 107.455 | | 3 | | 35.818 | |  |
| Uncorrected Total | | 15858.238 | | 6 | |  | |  |
| Corrected Total | | 3882.211 | | 5 | |  | |  |
| Dependent variable: Inhibition | | | | | | | |  |
| a. R squared = 1 - (Residual Sum of Squares) / (Corrected Sum of Squares) = .972. | | | | | | | |  |

IC_50_ is calculated from


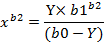


IC_50_ = 57.52 (μg/ml)

Figure 1: model fit with experimental values

Model 2: Nonlinear regression

Data 2: IC_50_ of Choloroform fraction against **α-amylase**

| Concentration (μg/ml) | 10 | 20 | 40 | 60 | 80 | 100 |
| --- | --- | --- | --- | --- | --- | --- |
| % Inhibition | 55.13 | 58.94 | 59.89 | 63.69 | 74.9 | 83.27 |

Model equation


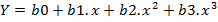


| **Parameter Estimates** | | | | |
| --- | --- | --- | --- | --- |
| Parameter | Estimate | Std. Error | 95% Confidence Interval | |
|  |  |  | Lower Bound | Upper Bound |
| b0 | 55.741 | 5.067 | 33.939 | 77.543 |
| b1 | .030 | .421 | -1.780 | 1.840 |
| b2 | .002 | .009 | -.036 | .040 |
| b3 | 8.007E-6 | .000 | .000 | .000 |

| **ANOVA^a^** | | | |
| --- | --- | --- | --- |
| Source | Sum of Squares | Df | Mean Squares |
| Regression | 26689.381 | 4 | 6672.345 |
| Residual | 10.991 | 2 | 5.495 |
| Uncorrected Total | 26700.372 | 6 |  |
| Corrected Total | 588.126 | 5 |  |
| Dependent variable: inhibition | | | |
| a. R squared = 1 - (Residual Sum of Squares) / (Corrected Sum of Squares) = .981. | | | |

IC_50_ is calculated from


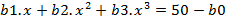


IC_50_= -246.3 (μg/ml)

Figure 2: model fit with experimental values

Model 3: Nonlinear regression

Data 3: IC_50_ of Ethanolic fraction against alpha amylase

| Concentration (μg/ml) | 10 | 20 | 40 | 60 | 80 | 100 |
| --- | --- | --- | --- | --- | --- | --- |
| % Inhibition | 49.05 | 60.65 | 62.93 | 71.86 | 80.8 | 82.89 |

Model equation


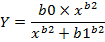


| **Parameter Estimates** | | | | |
| --- | --- | --- | --- | --- |
| Parameter | Estimate | Std. Error | 95% Confidence Interval | |
|  |  |  | Lower Bound | Upper Bound |
| b0 | 903.290 | 18999.780 | -59562.492 | 61369.071 |
| b1 | 1354096.385 | 150824089.703 | -478635470.637 | 481343663.408 |
| b2 | .242 | .400 | -1.032 | 1.516 |

| **ANOVA^a^** | | | |
| --- | --- | --- | --- |
| Source | Sum of Squares | Df | Mean Squares |
| Regression | 28570.589 | 3 | 9523.530 |
| Residual | 37.172 | 3 | 12.391 |
| Uncorrected Total | 28607.762 | 6 |  |
| Corrected Total | 839.276 | 5 |  |
| Dependent variable: inhibition^a^ | | | |
| a. R squared = 1 - (Residual Sum of Squares) / (Corrected Sum of Squares) = .956. | | | |

IC_50_ is calculated from


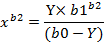


IC_50_ = 10.96 (μg/ml)

Figure 3: model fit with experimental values

Model 4: Nonlinear regression

Data 4: IC_50_ of *Uvaria chamae* against alpha amylase

| Concentration (μg/ml) | 10 | 20 | 40 | 60 | 80 | 100 |
| --- | --- | --- | --- | --- | --- | --- |
| % Inhibition | 34.6 | 42.78 | 50.78 | 57.6 | 68.25 | 74.71 |

Model equation


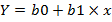


| **Parameter Estimates** | | | | |
| --- | --- | --- | --- | --- |
| Parameter | Estimate | Std. Error | 95% Confidence Interval | |
|  |  |  | Lower Bound | Upper Bound |
| b0 | 32.399 | 1.329 | 28.709 | 36.089 |
| b1 | .433 | .022 | .373 | .494 |

| **ANOVA^a^** | | | |
| --- | --- | --- | --- |
| Source | Sum of Squares | Df | Mean Squares |
| Regression | 19151.634 | 2 | 9575.817 |
| Residual | 11.670 | 4 | 2.917 |
| Uncorrected Total | 19163.303 | 6 |  |
| Corrected Total | 1153.830 | 5 |  |
| Dependent variable: inhibition^a^ | | | |
| a. R squared = 1 - (Residual Sum of Squares) / (Corrected Sum of Squares) = .990. | | | |

IC_50_ is calculated from


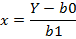


IC_50_= 40.64 (μg/ml)

Figure 4: model fit with experimental values

Model 5: Nonlinear regression

Data 5: IC_50_ of Acarbose against alpha amylase

| Concentration (μg/ml) | 10 | 20 | 40 | 60 | 80 | 100 |
| --- | --- | --- | --- | --- | --- | --- |
| % Inhibition | 57.41 | 72.43 | 75.67 | 77.95 | 79.09 | 81.94 |

Model equation


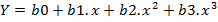


| **Parameter Estimates** | | | | | | | | |
| --- | --- | --- | --- | --- | --- | --- | --- | --- |
| Parameter | Estimate | | Std. Error | | 95% Confidence Interval | | | |
|  |  |  |  |  | Lower Bound | | Upper Bound | |
| b0 | 45.017 | | 7.274 | | 13.721 | | 76.314 | |
| b1 | 1.682 | | .604 | | -.917 | | 4.280 | |
| b2 | -.027 | | .013 | | -.082 | | .028 | |
| b3 | .000 | | .000 | | .000 | | .000 | |
| **ANOVA^a^** | | | | | | | |  |
| Source | | Sum of Squares | | Df | | Mean Squares | |  |
| Regression | | 33290.909 | | 4 | | 8322.727 | |  |
| Residual | | 22.647 | | 2 | | 11.324 | |  |
| Uncorrected Total | | 33313.556 | | 6 | |  | |  |
| Corrected Total | | 384.996 | | 5 | |  | |  |
| Dependent variable: inhibition | | | | | | | |  |
| a. R squared = 1 - (Residual Sum of Squares) / (Corrected Sum of Squares) = .941. | | | | | | | |  |

IC_50_ is calculated from


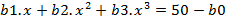


IC_50_=3.1185 (μg/ml)

Figure 5: model fit with experimental values

Model 6: Nonlinear regression

Data 6: IC_50_ of Ethyl acetate against alpha glucosidase

| Concentration (μg/ml) | 10 | 20 | 40 | 60 | 80 | 100 |
| --- | --- | --- | --- | --- | --- | --- |
| % Inhibition | 28.78 | 38.78 | 44.44 | 74.75 | 77.27 | 81.82 |

Model equation


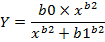


| **Parameter Estimates** | | | | |
| --- | --- | --- | --- | --- |
| Parameter | Estimate | Std. Error | 95% Confidence Interval | |
|  |  |  | Lower Bound | Upper Bound |
| b0 | 2502.376 | 118654.868 | -375110.370 | 380115.122 |
| b1 | 74274.803 | 7643127.327 | -24249567.520 | 24398117.125 |
| b2 | .507 | .568 | -1.299 | 2.313 |

| **ANOVA^a^** | | | |
| --- | --- | --- | --- |
| Source | Sum of Squares | Df | Mean Squares |
| Regression | 22377.901 | 3 | 7459.300 |
| Residual | 181.917 | 3 | 60.639 |
| Uncorrected Total | 22559.818 | 6 |  |
| Corrected Total | 2625.601 | 5 |  |
| Dependent variable: inhibition | | | |
| a. R squared = 1 - (Residual Sum of Squares) / (Corrected Sum of Squares) = .931. | | | |

IC_50_ is calculated from


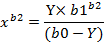


IC_50_ = 34.38 (μg/ml)

Figure 6: model fit with experimental values

Model 7: Nonlinear regression (logarithmic linear)

Data 7: IC_50_ of Chloroform fraction against alpha glucosidase

| Concentration (μg/ml) | 10 | 20 | 40 | 60 | 80 | 100 |
| --- | --- | --- | --- | --- | --- | --- |
| % Inhibition | 40.4 | 42.93 | 43.94 | 63.64 | 67.17 | 73.23 |

Model equation


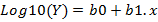


| **Parameter Estimates** | | | | |
| --- | --- | --- | --- | --- |
| Parameter | Estimate | Std. Error | 95% Confidence Interval | |
|  |  |  | Lower Bound | Upper Bound |
| b0 | 1.567 | .028 | 1.489 | 1.645 |
| b1 | .003 | .000 | .002 | .004 |

| **ANOVA^a^** | | | |
| --- | --- | --- | --- |
| Source | Sum of Squares | Df | Mean Squares |
| Regression | 18.009 | 2 | 9.005 |
| Residual | .005 | 4 | .001 |
| Uncorrected Total | 18.014 | 6 |  |
| Corrected Total | .065 | 5 |  |
| Dependent variable: loginhibition^a^ | | | |
| a. R squared = 1 - (Residual Sum of Squares) / (Corrected Sum of Squares) = .920. | | | |

IC_50_ is calculated from


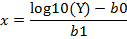


IC_50_ = 43.99 (μg/ml)

Figure 7: model fit with experimental values

Model 8: Nonlinear regression

Data 8: IC_50_ of Ethanolic fraction against alpha glucosidase

| Concentration (μg/ml) | 10 | 20 | 40 | 60 | 80 | 100 |
| --- | --- | --- | --- | --- | --- | --- |
| % Inhibition | 64.14 | 64.14 | 68.18 | 73.23 | 78.79 | 83.84 |

Model equation


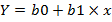


| **Parameter Estimates** | | | | |
| --- | --- | --- | --- | --- |
| Parameter | Estimate | Std. Error | 95% Confidence Interval | |
|  |  |  | Lower Bound | Upper Bound |
| b0 | 60.198 | .924 | 57.632 | 62.765 |
| b1 | .229 | .015 | .187 | .272 |

| **ANOVA^a^** | | | |
| --- | --- | --- | --- |
| Source | Sum of Squares | Df | Mean Squares |
| Regression | 31470.389 | 2 | 15735.195 |
| Residual | 5.645 | 4 | 1.411 |
| Uncorrected Total | 31476.034 | 6 |  |
| Corrected Total | 325.937 | 5 |  |
| Dependent variable: inhibition^a^ | | | |
| a. R squared = 1 - (Residual Sum of Squares) / (Corrected Sum of Squares) = .983. | | | |

IC50 is calculated from


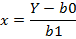


IC_50_= -44.53 (μg/ml)

Figure 8: model fit with experimental values

Model 9: Nonlinear regression

Data 9: IC_50_ of Uvaria chamae (crude) extract against alpha glucosidase

| Concentration (μg/ml) | 10 | 20 | 40 | 60 | 80 | 100 |
| --- | --- | --- | --- | --- | --- | --- |
| % Inhibition | 20.2 | 63.64 | 66.67 | 71.21 | 73.74 | 75.76 |

Model equation


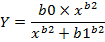


| **Parameter Estimates** | | | | |
| --- | --- | --- | --- | --- |
| Parameter | Estimate | Std. Error | 95% Confidence Interval | |
|  |  |  | Lower Bound | Upper Bound |
| b0 | 72.196 | 1.934 | 66.041 | 78.350 |
| b1 | 12.548 | .757 | 10.141 | 14.956 |
| b2 | 4.105 | .808 | 1.534 | 6.675 |

| **ANOVA^a^** | | | |
| --- | --- | --- | --- |
| Source | Sum of Squares | Df | Mean Squares |
| Regression | 25110.253 | 3 | 8370.084 |
| Residual | 40.754 | 3 | 13.585 |
| Uncorrected Total | 25151.008 | 6 |  |
| Corrected Total | 2183.626 | 5 |  |
| Dependent variable: inhibition | | | |
| a. R squared = 1 - (Residual Sum of Squares) / (Corrected Sum of Squares) = .981. | | | |

IC_50_ is calculated from


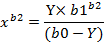


IC_50_ = 15.29 (μg/ml)

Figure 9: model fit with experimental values

Model 10: Nonlinear regression

Data 10: IC_50_ of Acarbose against alpha glucosidase

| Concentration (μg/ml) | 10 | 20 | 40 | 60 | 80 | 100 |
| --- | --- | --- | --- | --- | --- | --- |
| % Inhibition | 16.67 | 58.08 | 67.17 | 68.69 | 69.7 | 81.31 |

Model equation


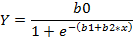


| **Parameter Estimates** | | | | |
| --- | --- | --- | --- | --- |
| Parameter | Estimate | Std. Error | 95% Confidence Interval | |
|  |  |  | Lower Bound | Upper Bound |
| b0 | 71.760 | 3.244 | 61.437 | 82.083 |
| b1 | -3.817 | 1.169 | -7.537 | -.097 |
| b2 | .263 | .079 | .011 | .514 |

| **ANOVA^a^** | | | |
| --- | --- | --- | --- |
| Source | Sum of Squares | Df | Mean Squares |
| Regression | 24225.565 | 3 | 8075.188 |
| Residual | 125.141 | 3 | 41.714 |
| Uncorrected Total | 24350.706 | 6 |  |
| Corrected Total | 2555.869 | 5 |  |
| Dependent variable: inhibition | | | |
| a. R squared = 1 - (Residual Sum of Squares) / (Corrected Sum of Squares) = .951. | | | |

IC50 is calculated from


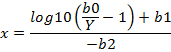


IC_50_ = 15.89 (μg/ml)

Figure 10: model fit with experimental values

IC_50_ values of α-amylase and α-glucosidase inhibition by the crude extract of *Uvaria chamae*and its fractions

| **Extract/ Fractions** | **IC_50_(μg/ml)** | |
| --- | --- | --- |
|  | Alpha-amylase | Alpha-glucosidase |
| *Uvaria chamae* | 40.64 | 15.29 |
| Ethyl acetate | 57.52 | 34.38 |
| Choloroform | ˗246.3 | 43.99 |
| Ethanol | 10.96 | -44.53 |
| Acarbose | 3.12 | 15.89 |
